# The Boston Veterinary Institute

**Published:** 1855-09

**Authors:** 


### THE BOSTON VETERINARY INSTITUTE.

It is now more than ten years since a Veterinary College was talked of by the Massachusetts Agricultural Society. As early as 1846 a young gentleman was sent under their auspices to study the Diseases of the Horse, at Alfort, near Paris. This young physician, whom we knew well, and who was endowed with a very high order of talents, prosecuted his studies to their completion and returned to the United States in 1848. It was announced soon after, that he would give a series of lectures on Veterinary Medicine in Boston, but before he had prepared the introductory to the course, he came to a sudden and melancholy end. The project of the school, however, was not entirely abandoned, and after the lapse of nearly seven years it appears under the name of the Boston Veterinary Institute. Concerning the ends and aims and prospects of the enterprise the *Boston Med. and Sur. Journal* makes the following remarks:

"The importance of an institution which should be able to disseminate sound instruction on the subject of veterinary medicine, and supply the community with a class of competent veterinary surgeons, will be at once acknowledged, when it is remembered to how great a degree we are dependent upon domesticated animals for our pleasure, our support, and our wealth. Like the human species they are subject to a great variety of maladies, which can only be efficiently controlled or relieved by a thorough acquaintance with their anatomy, physiology, pathology, and hygiene, and with the remedies best adapted to the cure of their diseases. The amount of ignorance which prevails, in this country at least, upon the subject, is very great, and yet it is but little appreciated, even by those who are most likely to suffer from it. The most valuable animals, when sick, are frequently confided to the care of horse-doctors and cattle-doctors, who are as ignorant of the principles of veterinary medicine as they are rash and unskillful in practice. With a few exceptions, this class of practitioners, with us, have had no regular education to qualify them for the exercise of a profession which requires in some respects more knowledge, as well as a higher sagacity, than is called for in the treatment of the human patient; for the physician is deprived of a most important source of information, both in the detection of symptoms, and in the effect of remedies, from the incapacity of the sufferer to describe his own sensations.

"Incompetent, as too many of our veterinary surgeons are, we believe that, even in its depressed condition among us, the profession yields a handsome return to those engaged in its practice, and there is no doubt that surgeons, properly qualified by a regular course of study at some institution of known reputation, would find a rich field for the exercise of their art in our community, where valuable animals are often sacrificed, either in consequence of the ignorance of the doctor, or from the skepticism of the owner, who in despair refuses all medical interference. The profession has hitherto been looked upon as rather beneath the notice of an educated and cultivated man, though upon what grounds we are at a loss to conceive. In order to become accomplished in it, one must spend years in patient study, in attendance on lectures and clinical instruction, and in dissection. He should be familiar, to some extent, at least, with all the different branches which are required for the ordinary practitioner of medicine and surgery; and an acquaintance with those departments of science which have no immediate bearing upon veterinary medicine, will tend indirectly, by promoting habits of observation and investigation, to qualify him for the study and treatment of the maladies of the brute creation. In England and France there are several schools for instruction in this branch of knowledge, which offer every advantage that can be desired. That at Alfort, near Paris, has long been celebrated. It contains about 300 students, and the course of study, which extends through four years, embraces lectures on anatomy, chemistry, botany, materia medica, and pharmacy, veterinary surgery, with operations, and the practice of medicine as applied to animals. To use the forge is also taught.

"We are glad to see that there is a prospect that this subject will receive among us that attention which it has so long needed. In May last, an act of legislature was passed, incorporating the 'Boston Veterinary Institute,' the object of which is to afford ample instruction to persons desirous of qualifying themselves for the practice of veterinary medicine and surgery. The plan of instruction includes lectures on the Anatomy of Physiology of the Horse, on Theory and Practice of Veterinary Medicine and Surgery, and on Cattle Pathology. Students will also be allowed to attend the lectures on Chemistry and Pathological Anatomy in the medical department of Harvard University, and Clinical Lectures will be given by the Faculty.

"We hope that this effort in behalf of a noble and useful purpose, will meet with a corresponding encouragement from the community. Without assistance at this early period of its organization, the School will not be able to sustain itself. All who own horses or stock, should contribute something towards an enterprise which will be a benefit to them. It is hoped that the next legislature will make the Institution a handsome grant. When money is so freely lavished on botanic colleges and female medical schools, it surely ought not to be withheld from an object whose practical utility will, we presume, be questioned by no one."

